# Why publish in *Biology Letters*?

**DOI:** 10.1098/rsbl.2022.0356

**Published:** 2022-09-14

**Authors:** David Beerling

**Affiliations:** *Editor-in-chief*

Competition for authors among broad spectrum journals covering evolutionary biology, ecology and global change biology is intense and increasing with the advent of new online-only journals. In such a publishing ecosystem, it is important to be clear why *Biology Letters* should be the stand-out journal of choice for potential authors.

Do you have a clear, concise scientific finding that you would like to present to biologists from various disciplines? Do you want to reach a wide audience quickly following a thorough and fair peer review from a Royal Society journal?

Consider publishing your new results in *Biology Letters*! We publish exciting papers of broad appeal to its wide readership. Emphasis is on rigorous study designs that allow clear robust conclusions to be drawn across the broadest range of biosciences. Working with a word limit of 2500 words for research and opinion piece manuscripts and 5000 words for reviews (excluding references, title pages and text within tables), our authors keep to the point, when reporting on their study design, results, limitations and wider significance. This *Biology Letters* template lends itself to powerful discoveries that can be articulated concisely in a small number of well-crafted paragraphs and one or two essential figures.

Our format is advantageous to readers and reviewers, as well as authors. It means readers can read the paper and get the information they need quickly without having to dig through a full-format article. It also means we have exceptionally fast review and publication times while maintaining high quality (our external peer review times are the fastest among all Royal Society journals). Rapid review times should, however, not be mistaken for a lack of rigour. The *Biology Letters* editorial process starts with a Board Member who recommends reviewers, and concludes with the Handling Editor who provides a decision based on the reviewer comments and the Board Member's appraisal. The process is fair and helps us attain a thoroughly vetted manuscript of the highest quality, with both biological science experts and methodologist editors working to edit submissions. Our excellent team of editors always seek to obtain highly qualified reviewers who can provide a timely and well-informed assessment of a submission.

We are instigating several new initiatives in the coming months which I'm delighted to highlight here. *Biology Letters* is (and always has been) strongly supportive of early career researchers (ECRs). We are excited to report that we will soon be launching an ECR best paper prize. Please check our Twitter feed for further information on this development. To further promote ECR activities, we are publishing a New Talent collection that will carry a range of papers illustrating the breadth and depth of ECR research activities. Look out for these papers in our Special Features page—https://royalsocietypublishing.org/rsbl/special-features—where you can also see all the Features we have published. Have an idea for a Special Feature that you would like to guest edit? Contact the editorial office at biologyletters@royalsociety.org with your proposal.

Owing in part to our regular media coverage of our excellent papers, we have attained our highest Impact Factor since our launch and we want to thank our contributing authors, the Editorial Board and referees for their advice, expertise and advocacy—it truly has made a real difference in the quality of the work we receive and publish. As signatories of the Declaration on Research Assessment (DORA), we recognize that other metrics exist and these can be found on our metrics page here: https://royalsocietypublishing.org/rsbl/citation-metrics.

The key to the success of *Biology Letters* is the efficiency of our editorial staff in handling, processing and publishing manuscripts. I would like to offer sincere thanks to our Publishing Editor, Surayya Johar, our Editorial Coordinators, Raminder Shergill and Charlotte Lindsay and our whole Production team for their efficient editing and production of the journal over the past 12 challenging months. We are grateful to all them for of their hard work. Importantly, we continue to thank all of our authors, readers, reviewers and the editorial board for their support of the journal over the past year that has contributed to our success.

